# Genetic risk and incident venous thromboembolism in middle‐aged and older adults following COVID‐19 vaccination

**DOI:** 10.1111/jth.15879

**Published:** 2022-10-05

**Authors:** Junqing Xie, Albert Prats‐Uribe, Maria Gordillo‐Marañón, Victoria Y. Strauss, Dipender Gill, Daniel Prieto‐Alhambra

**Affiliations:** ^1^ Centre for Statistics in Medicine, NDORMS University of Oxford Oxford UK; ^2^ Data Analytics and Methods Task Force European Medicines Agency Amsterdam Netherlands; ^3^ Institute of Cardiovascular Science University College London London UK; ^4^ Department of Epidemiology and Biostatistics, School of Public Health Imperial College London London UK; ^5^ Chief Scientific Office, Research and Early Development Novo Nordisk Copenhagen Denmark; ^6^ Medical Informatics Erasmus Medical Center University Rotterdam Netherlands

**Keywords:** ChAdOx1 nCoV‐19, COVID 19 vaccine, COVID‐19 vaccine Pfizer‐BioNTech, genetic predisposition to disease, venous thromboembolism

## Abstract

**Background:**

COVID‐19 vaccination has been associated with increased venous thromboembolism (VTE) risk. However, it is unknown whether genetic predisposition to VTE is associated with an increased risk of thrombosis following vaccination.

**Methods:**

Using data from the UK Biobank, which contains in‐depth genotyping and linked vaccination and health outcomes information, we generated a polygenic risk score (PRS) using 299 genetic variants. We prospectively assessed associations between PRS and incident VTE immediately after first‐ and the second‐dose vaccination and among historical unvaccinated cohorts during the pre‐ and early pandemic. We estimated hazard ratios (HR) for PRS‐VTE associations using Cox models.

**Results:**

Of 359 310 individuals receiving one dose of a COVID‐19 vaccine, 160 327 (44.6%) were males, and the mean age at the vaccination date was 69.05 (standard deviation [SD] 8.04) years. After 28‐ and 90‐days’ follow‐up, 88 and 299 individuals developed VTE, respectively, equivalent to an incidence rate of 0.88 (95% confidence interval [CI] 0.70–1.08) and 0.92 (0.82–1.04) per 100 000 person‐days. The PRS was significantly associated with a higher risk of VTE (HR per 1 SD increase in PRS, 1.41 (1.15–1.73) in 28 days and 1.36 (1.22–1.52) in 90 days). Similar associations were found in the historical unvaccinated cohorts.

**Conclusions:**

The strength of genetic susceptibility with post‐COVID‐19‐vaccination VTE is similar to that seen in historical data. Additionally, the observed PRS‐VTE associations were equivalent for adenovirus‐ and mRNA‐based vaccines. These findings suggest that, at the population level, the VTE that occurred after the COVID‐19 vaccination has a similar genetic etiology to the conventional VTE.

## Essentials

- Venous thromboembolism (VTE) after COVID‐19 vaccination has been hypothesized as an immune‐mediated particular type. Little is known about genetic susceptibility to VTE among recipients of COVID‐19 vaccines.
- We analyzed a large prospective cohort of 359 310 participants from UK Biobank who received at least one dose of the vaccine.
- The association between a previously validated polygenic risk score and incident VTE after the first and the second‐dose vaccination was comparable to that seen in the historically unvaccinated population.
- These findings suggest that the post‐vaccination VTE and the conventional VTE have similar genetic architecture at the population level.

## 1 INTRODUCTION

Venous thromboembolism (VTE), primarily comprising deep vein thrombosis and pulmonary embolism, is predominantly a disease of older age that affects nearly 10 million people worldwide every year and frequently leads to morbidities and death.^1^, ^2^, ^3^ SARS‐CoV‐2 infection and COVID‐19 have been recognized as novel environmental triggers for VTE. Also, a number of spontaneous thromboembolic complications were reported after adenovirus vector COVID‐19 vaccination,^4^ prompting the withdrawal of the Oxford‐AstraZeneca vaccine (ChAdOx1) from several markets or the imposition of restrictions on its use.^5^ *In vitro* studies have shown PF4‐dependent platelet activation in patients developing thromboembolic events following vaccination with adenovirus vector vaccines.^6^ Such PF4‐dependent platelet activation is also observed during the development of rare vaccine‐induced immune thrombotic thrombocytopenia,^7^ although observational evidence has later emerged suggesting that VTE risks are substantially higher after SARS‐CoV‐2 infection than after vaccination, regardless of vaccine type or brand.^8^

Twins and family studies have shown that VTE is highly heritable, and a few clinical studies suggest that inherited thrombophilia can interact with various environmental risk factors, such as infectious pneumonia.^9^, ^10^ Additionally, many common genetic variants associated with VTE and their effect sizes have been identified in large‐scale genome‐wide association studies (GWASs), making it possible to construct a polygenic risk score (PRS) to quantify genetic predisposition to the VTE trait.

The present study aimed to assess the association between a previously validated PRS for conventional VTE and the post‐COVID‐19‐vaccination VTE, where thrombotic events following COVID‐19 vaccination were hypothesized to be involved in distinctive pathobiological mechanisms.

## 2 METHODS

### 2.1 UK Biobank

The UK Biobank (UKBB) is a prospective cohort of more than 500 000 individuals recruited from England (89%), Wales (7%), and Scotland (4%) between 2006 and 2010. Age at baseline enrollment ranged from 40 to 69 years. Comprehensive information on demographics, socioeconomics, lifestyle factors, physical metrics, and medical history were collected using a computer‐based questionnaire and a standardized portfolio of measurements.^11^ Genome‐wide genotyping was performed using two closely related purpose‐designed arrays (the UK BiLEVE Axiom array and UK Biobank Axiom array). The genetic data have been quality controlled as described in previous studies.^12^ Over the follow‐up, health‐related outcomes were captured through linkage to external data sources, including primary care, hospital inpatient, and death data. Additional information is available at https://www.ukbiobank.ac.uk/.

UKBB received ethical approval from the research ethics committee (National Health Service's National Research Ethics Service North West (11/NW/0382)), with all participants providing written consent. This study was conducted under Application Number 65397.

### 2.2 Study population and design

For the vaccinated cohorts, all UKBB participants from England who received at least one dose of BNT162b2 or ChAdOx1COVID‐19 vaccines between December 2, 2020 (i.e., vaccines approval date in the UK), and September 31, 2021, were included. Eligible participants were followed from the vaccination date (index date) to outcome, death, or the end of prespecified follow‐up windows, whichever came first. The participants from Wales or Scotland were not included because of the lack of linkage to their vaccination records at the time of this analysis performed.

Two historical unvaccinated cohorts (named early‐pandemic and prepandemic cohorts) were constructed for comparison. For the early‐pandemic cohort, the observational period started from March 23, 2020 (the announcement of the first national lockdown in the United Kingdom, index date) to December 1, 2020 (the last day before COVID‐19 vaccines approval). In contrast, the prepandemic cohort was followed 1 year earlier, from March 23, 2019 (index date), to March 23, 2020. In addition, a COVID‐19 infection cohort was curated with the date of infection as index date where the infection was confirmed based on polymerase chain reaction–positive testing results obtained through linkage to the Public Health England's Second Generation Surveillance System.^13^ People with historical VTE at the study entry date were excluded for all study cohorts.

### 2.3 Polygenic risk score

We derived polygenic risk scores (PRS) for VTE as a weighted sum of risk alleles, using summary statistics of 297 single nucleotide polymorphisms (SNPs) from a GWAS on VTE,^14^ and additionally included the two clinically validated mutations: factor V Leiden p.R506Q and prothrombin G20210A to maximize the PRS predictive power and its quantitative impact.^15^ Given that the selected GWAS sample included UKBB participants, we conducted a sensitivity analysis using a newly generated alternative PRS based on a meta‐analysis of 12 GWASs that did not cover UKBB participants.^16^ We standardized the continuous PRS by *z*‐transformation to achieve a zero mean and standard deviation of 1 based on the entire UKBB population.

Details on data manipulation and completed lists of SNPs included in the primary PRS and alternative PRS are provided in the Appendix S1.

### 2.4 Vaccination against COVID‐19

In the UK, vaccination information for all residents who registered with a general practitioner (GP) has been directly or indirectly added to patient's GP medical records within 48 hours.^17^ Specifically, vaccination status for UKBB participants was obtained from the linked primary care records provided by the two GP system suppliers: EMS and TPP (latest update: September 31, 2021). The clinical codes used for the first and second dose of the COVID‐19 vaccines were “1324681000000101*”* and “1324691000000104” in EMS (SNOMED CT) and “Y29e7” and “Y29e8” in TPP (READ v3), respectively.

### 2.5 Venous thromboembolism

Incident VTE, including pulmonary embolism, deep vein thrombosis, and superficial thromboembolism such as thrombophlebitis of lower extremities and unusual site thrombosis, was captured within 28 and 90 days after the index date using linked hospital admission data from Hospital Episode Statistics, which contains all admissions in National Health Service hospitals in England. Mortality was ascertained from linked national death registry data. We used the earliest date of VTE diagnosis as the event date. The same International Classification of Diseases‐10 codes were used to identify VTE outcome for all study cohorts and are listed in Appendix S1.

### 2.6 Statistical analyses

We used Cox proportional‐hazards models to assess the associations between the PRS and VTE outcome. We computed hazard ratios (HR) and their 95% confidence intervals (CI) with adjustment for age (at the index date), sex, and genetic ancestry (quantified by the first 10 principal components). To identify the high genetic risk group, we tested three cutoff quantiles of PRS separately, including upper tertile (top 33%), quintile (top 20%), and the top 5% with the lower 66% as the reference. To ensure sufficient statistical power, this analysis was only performed in the 90‐day follow‐up window. We evaluated the balance of baseline characteristics within each comparison pair according to a list of prespecified covariates and adjusted for them in the Cox model if their absolute standardized mean difference was greater than 0.1. Considering varying VTE rates across the reference groups, we derived absolute risk increases (ARI) between high‐risk and the reference PRS categories using the formula: (adjusted HR – 1) × cumulative incidence in the reference group.

We calculated HRs for diabetes as a negative control outcome to examine the specificity of the PRS and the likelihood of potential residual confounding. Diabetes was chosen with considerations that it is a well‐developed disease phenotype and not biologically related to the VTE PRS. In a subcohort where the EMIS system provided the primary care data, and vaccine types were recorded, separate HRs were estimated among either ChAdOx1 or BNT162b2 vaccine recipients. Given that the heterologous prime‐boost vaccination schedule in the United Kingdom is very uncommon^18^ (with <1% in our data), no specific analyses in this regard have been performed.

All the analyses were performed using PLINK1.9, QCTOOL v2, and R 4.1.2 software.

## 3 RESULTS

### 3.1 Characteristics of vaccine recipients in UKBB

**FIGURE 1:**
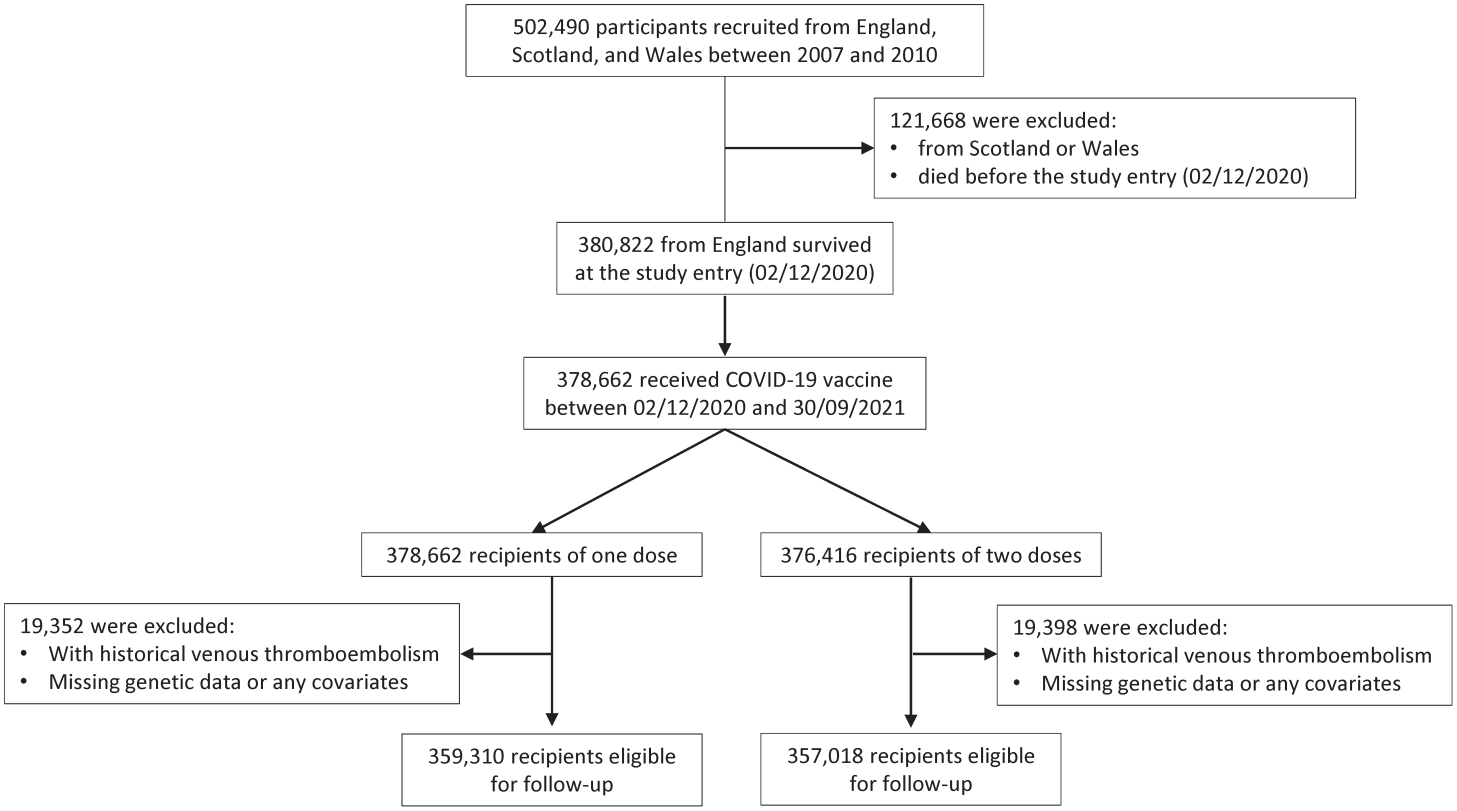
Flow chart of the study selection process.

Of 380 822 UKBB participants eligible at the study entry (December 2, 2020), 378 662 (99.4%) and 376 416 (98.8%) received the first and second dose of COVID‐19 vaccines, respectively, until the study end date (September 31, 2021) (Figure 1). For the one‐dose cohort, the mean age was 69.05 years (standard deviation 8.04), and 160 327 (44.6%) were male (Table 1). A similar demographic profile was observed for the two‐dose cohort (Table 1). The PRS approximated a normal distribution within each cohort (Appendix S1).

**TABLE 1:** Baseline characteristics by the genetic risk categories (one dose)

	Overall	High PRS groups		
		Top 33%	Top 20%	Top 5%
Number	359 310	119 770	71 862	17 965
Demographics				
Age, mean (SD)	69.05 (8.04)	69.10 (8.01)	69.14 (8.02)	69.11 (8.02)
Sex, male (%)	160 327 (44.6)	53 178 (44.4)	31 766 (44.2)	7909 (44.0)
BMI, mean (SD)	27.30 (4.69)	27.29 (4.69)	27.29 (4.69)	27.26 (4.69)
Socioeconomic status, mean (SD)				
Indices of multiple deprivation	17.18 (13.62)	17.00 (13.50)	16.91 (13.43)	16.71 (13.27)
Income score	0.11 (0.09)	0.11 (0.09)	0.11 (0.09)	0.11 (0.09)
Employment score	0.09 (0.06)	0.09 (0.06)	0.09 (0.06)	0.08 (0.06)
Health score	−0.11 (0.84)	−0.12 (0.84)	−0.13 (0.84)	−0.14 (0.84)
Education score	15.23 (15.84)	15.14 (15.75)	15.09 (15.74)	14.98 (15.70)
Housing score	19.76 (10.12)	19.64 (10.08)	19.58 (10.07)	19.47 (10.10)
Crime score	−0.06 (0.77)	−0.06 (0.77)	−0.07 (0.77)	−0.07 (0.78)
Living environmental score	18.28 (15.07)	18.15 (14.96)	18.08 (14.89)	17.93 (14.66)

*Note:* Indices of multiple deprivation offer a more complex and detailed view of deprivation, based on more factors than the Townsend index. All scores have been scaled to 0–1, 0–100, or even distributions standardized around 0, with higher values indicating more deprived. Details of individual score has been described in the GOV.UK (https://www.gov.uk/government/collections/english‐indices‐of‐deprivation).

### 3.2 Association of the PRS with incident VTE

During the follow‐up periods, 88 and 299 individuals developed VTE within 28 and 90 days after first‐dose vaccination (Table 2), equivalent to an incidence rate of 0.88 (95% CI 0.70–1.08) and 0.92 (95% CI 0.82–1.04) per 100 000 person‐days. The unadjusted and adjusted HRs for VTE associated with the primary PRS were similar, with the latter being 1.41 (95% 1.15–1.73) per 1‐SD increase in PRS (1‐SD PRS) over 28‐day follow‐up and 1.36 (95% 1.22–1.52) over 90 days. The association between the PRS value and risk of VTE appears to be monotonic in nature (Appendix S1). After the second dose vaccination, the association between PRS and VTE was slightly attenuated (HR: 1.30 [95% 1.04–1.61] per 1‐SD PRS and 1.33 [95% 1.18–1.49] in the 28‐ and 90‐day' follow‐up window, respectively) (Table 2). Although there was a seemingly inverted U‐shaped relationship between the PRS and estimate of VTE risk following the second dose of vaccine, wide CIs limit the reliability of this finding.

**TABLE 2:** Association between the genetic score and incident venous thromboembolism in vaccinated and reference cohorts

	Number of people	Number of cases	Incidence rate (95% CI)^a^	Primary PRS		Sensitivity PRS
				Unadjusted hazard ratio (95% CI)^b^	Adjusted hazard ratio (95% CI)^b^	Adjusted hazard ratio (95% CI)^b^
Vaccinated cohorts						
28 days after one dose	359 310	88	0.88 (0.70–1.08)	1.41 (1.15–1.73)	1.41 (1.15–1.73)	1.38 (1.13–1.70)
90 days after one dose	359 310	299	0.92 (0.82–1.04)	1.36 (1.22–1.52)	1.36 (1.22–1.52)	1.34 (1.20–1.50)
28 days after two doses	357 018	78	0.78 (0.62–0.97)	1.31 (1.05–1.63)	1.30 (1.04–1.61)	1.25 (1.00–1.55)
90 days after two doses	357 018	269	0.83 (0.74–0.96)	1.34 (1.19–1.50)	1.33 (1.18–1.49)	1.29 (1.15–1.46)
Historically unvaccinated cohorts						
Whole UKBB (prepandemic)	391 752	1078	0.76 (0.71–0.80)	1.36 (1.29–1.45)	1.36 (1.28–1.44)	1.34 (1.26–1.42)
Whole UKBB (early pandemic)	387 829	846	0.80 (0.74–0.85)	1.35 (1.26–1.44)	1.34 (1.26–1.44)	1.27 (1.19–1.36)
Infected cohorts						
28 days after infection	24 700	155	25.6 (21.8–30.0)	1.29 (1.10–1.51)	1.32 (1.13–1.55)	1.32 (1.12–1.55)
90 days after infection	24 700	186	10.9 (9.4–12.6)	1.27 (1.10–1.46)	1.29 (1.12–1.49)	1.28 (1.11–1.48)

*Note:* The prepandemic was defined as the period between March 23, 2019, and March 23, 2020. The early pandemic was defined as the period between March 23, 2020, and December 1, 2020. The negative control outcome was incident diabetes. Abbreviations: PRS, polygenic risk score; UKBB, UK Biobank.

^a^ Per 100 000 person‐days.

^b^ Per 1‐SD increase of PRS.

The observed rates and effect sizes of the observed associations were similar when comparing the vaccinated and historical (unvaccinated) cohorts, demonstrating that genetic susceptibility to postvaccination VTE was not different to that related to any other VTE seen in the general population. Also, although absolute incidence rates of VTE in the infected cohort were substantially higher than those in other cohorts, the PRS‐VTE association persisted. A sensitivity analysis using an alternative PRS found similar although slightly weaker associations (Table 2).

Finally, no associations were observed for our proposed negative control outcome: the HR between PRS and incident diabetes was 1.02 (95% 0.98–1.06) in the prepandemic and 0.98 (95% 0.93–1.04) in the early pandemic period (Appendix S1).

### 3.3 Identification of high‐risk group

**FIGURE 2:**
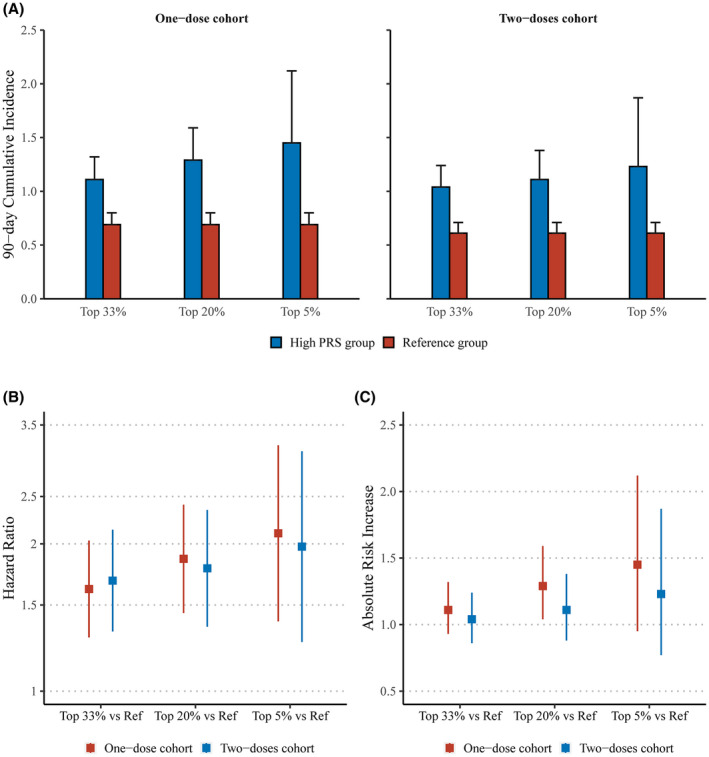
Ninety‐day cumulative incidence (A), hazard ratios (B), and absolute risk increases (C) of three predefined high genetic risk groups vs the reference. Reference: participants with lower 66% PRS. Hazard ratios and absolute risk increases were calculated in comparison with the reference group.

Figure 2 presents HRs and ARI for VTE across three predefined high‐risk categories. Briefly, relative risks increased with cutoffs from 33% to 5%, corresponding to HRs ranging from 1.67 (95% CI 1.33–2.09) to 2.10 (95% CI 1.39–3.18) in the one‐ and from 1.66 (95% CI 1.30–2.11) to 1.97 (95% CI 1.26–3.09) in the two‐dose cohorts. Also, there was a linear increasing trend for absolute risk differences, with ARI of 0.45 (95% CI 0.22–0.74) to 0.76 (95% CI 0.27–1.51) and 0.40 (95% CI 0.19–0.67) to 0.59 (95% CI 0.16–1.28) in the one‐ and two‐dose cohort, respectively.

### 3.4 Different vaccine types

Among 221 875 recipients with vaccine‐type information available (138 059 received ChAdOx1 and 83 816 received BNT162b2), the observed PRS‐VTE associations were similar across each dose and follow‐up window: HR ranged from 1.24 (95% CI 0.88–1.77) to 1.63 (95% CI 1.34–1.98) in ChAdOx1 vaccinated cohorts, and from 1.20 (95% CI 0.82–1.76) to 1.38 (95% CI 0.99–1.93) in BNT162b2 vaccinated people (Table 3). Noticeably, the background VTE incidence rates in BNT162b2 vaccinated cohorts were almost doubly higher than those in the ChAdOx1 vaccinated one, which was expected given that the former vaccine was approved earlier in the UK and prioritized for older and more vulnerable populations.^19^

**TABLE 3:** Exploratory analyses for different vaccine types

Cohorts	ChAdOx1			BNT162b2		
	No. of cases/people	Incidence rate (95% CI)^a^	Adjusted hazard ratio (95% CI)^b^	No. of cases/people	Incidence rate (95% CI)^a^	Adjusted hazard ratio (95% CI)^b^
28 days after one dose	25/138 059	0.65 (0.42–0.96)	1.45 (0.99–2.13)	33/83 816	1.41 (0.97–1.98)	1.38 (0.99–1.93)
90 days after one dose	94/138 059	0.76 (0.61–0.93)	1.63 (1.34–1.98)	99/83 816	1.31 (1.07–1.60)	1.24 (1.02–1.50)
28 days after two doses	30/136 002	0.79 (0.53–1.13)	1.24 (0.88–1.77)	26/80 709	1.15 (0.75–1.69)	1.20 (0.82–1.76)
90 days after two doses	100/136 002	0.82 (0.67–1.00)	1.53 (1.27–1.85)	87/80 709	1.20 (0.96–1.48)	1.22 (1.00–1.51)

^a^ PER 100 000 person‐days.

^b^ PER 1‐SD increase of PRS.

## 4 DISCUSSION

Our study showed that a PRS for conventional VTE could identify people at increased risk of VTE within 28 or 90 days after receiving one or two doses of COVID‐19 vaccines. Furthermore, the strength of the PRS association to post‐COVID‐19 vaccination VTE was similar to that seen for VTE before COVID‐19 vaccination rollout. Taken together, we found no evidence of a potential interaction between COVID‐19 vaccination and human genetic variations on VTE risk at the population level.

The PRS used in the present study was developed and validated by Klarin et al. The study found a 2.5‐ to three‐fold increased risk of VTE associated with the highest 5% of the score in both case–control and prospective cohort study settings.^14^ Recently, Marston et al. tested the performance of the PRS among cardiometabolic disease patients to predict VTE and observed a similar magnitude of effect (2.7‐fold for top 33% vs bottom 66%).^20^ Despite being aligned with these findings, the PRS‐VTE associations estimated in our study were consistently weaker than the previously reported ones even after the incorporation of the two clinically validated variants, possibly because of the discrepancies in defining VTE phenotypes between the original score deviation and this validation study. Also, because our cohort only consisted of VTE‐naïve and relatively older participants, those with higher genetic risk might have had a VTE in their earlier age and thus been excluded. As expected, our PRS was not associated with the proposed negative control outcome (incident diabetes), to some extent, demonstrating its specificity for VTE prediction.

The results of this study support several noteworthy conclusions. First, our data showed that individuals' genetic susceptibility to VTE was a risk factor for VTE among the COVID‐19‐vaccinated population. Second, this genetic risk was independent of traditional risk factors such as old age, obesity, and comorbidity, as indicated by no associations between the PRS and baseline characteristics (Table 1). Third, by designing a historical comparison arm in the same population, our data suggest that clinically significant interactions between individuals' genetic background and COVID‐19 vaccination are unlikely, which has particular implications for patients with hereditary VTE predisposing traits who are hesitant to be vaccinated because of concerns regarding related recent vaccine safety signals. Fourth, we identified 5% of people with more than two‐fold higher VTE risk by using this genetic score, it should be of public health relevance and can inform potential intervention policies given the absolute size of COVID‐19‐vaccinated population. Our analyses have some potential limitations. First, VTE often presents variable clinical manifestations with challenging differential diagnoses such as myocardial infarction and congestive heart failure.^2^ Consequently, identification of VTE in a real‐world setting is likely subject to information bias, which typically drives risk estimates towards the null. Second, we were not able to generate a parallel unvaccinated comparison group because more than 99% of UKBB participants had been vaccinated. However, we constructed a historical comparison cohort with similar characteristics to those vaccinated. Also, given the relatively short follow‐up after vaccination, the long‐term impact of the genetic factor remains to be determined. Third, although we also constructed a secondary PRS for VTE, the weights of each included SNP have not been previously validated, and their utility in a PRS remains unknown. Opportunely, it conferred consistent results as the primary PRS did, likely because both PRSs included the factor V Leiden p.R506Q and prothrombin G20210A variants, which are known causes of inherited thrombophilia predisposing to acute thrombotic syndromes.^21^, ^22^ Fourth, risk estimates in our study for each vaccine type should be considered exploratory in nature because of evident differences in the baseline risk for VTE seen between people vaccinated with the two vaccines and the lack of evidence on post‐vaccination VTE associated with mRNA vaccines. Last, the generalizability of our findings should be tested in more diverse ethnic populations as more integrated data sources containing in‐depth genetic, vaccination, and health information becomes available.

This study benefits from the use of a large prospective cohort with comprehensive genetic, COVID‐19 vaccination, COVID‐19 infection status, and VTE phenotype data linked at the individual level, the application of the state‐of‐the‐art PRS, and robust analytic methods by designing multiple comparison groups and a negative control outcome. To our knowledge, this is the first study to show that individuals who developed post‐COVID‐19 vaccination VTE had a genetic predisposition to VTE, and that the association between the genetic risk factors and post‐COVID‐19 vaccination VTE is similar to the association with conventional VTE.

## 5 CONCLUSIONS

A published PRS for VTE, constructed using common genetic variants with small effects on VTE, was associated with increased VTE risk following COVID‐19 vaccination. This association was similar to that seen historically, both in prepandemic times and during the first year of the COVID‐19 pandemic, before vaccines were available. Our data do not support a clinically meaningful interplay between genetic predisposition and COVID‐19 vaccines on the occurrence of VTE events. These findings suggest that the clinical management of VTE among the vaccinated population should not be disturbed by the concern of gene–vaccine interaction, and that people at high genetic risk of VTE such as those with inherited thrombophilia might have a modest excess risk of VTE occurrence following vaccination.

## AUTHOR CONTRIBUTIONS

D.P.A., J.Q.X., and D.G. were responsible for the study design. J.Q.X. did the data analyses, and A.P.U. checked the statistical codes. J.Q.X. and D.P.A. drafted the manuscript, and all coauthors reviewed and approved it for submission.

## CONFLICT OF INTEREST

D.P.A.’s research group has received grants and advisory or speaker fees from Amgen, Astellas, AstraZeneca, Chiesi‐Taylor, Johnson & Johnson, and UCB; and Janssen, on behalf of Innovative Medicines Initiative–funded European Health Data Evidence Network and European Medical Information Framework consortiums and Synapse Management Partners, have supported training programs, open to external participants, organized by his department. D.G. is employed part‐time by Novo Nordisk. J.X., A.P.U., M.G.M., and V.Y.S. declare no conflicts of interest.

## FUNDING INFORMATION

This study was funded by the European Medicines Agency (EMA/2018/21/PE). J.X. is funded through Jardine‐Oxford Graduate Scholarship and a titular Clarendon Fund Scholarship. D.G. is supported by the British Heart Foundation Research Centre of Excellence (RE/18/4/34215) at Imperial College London and by a National Institute for Health Research Clinical Lectureship (CL‐2020‐16‐001) at St. George's, University of London. D.P.A. is funded through an NIHR Senior Research Fellowship (grant SRF‐2018‐11‐ST2‐004) and received partial support from the Oxford NIHR Biomedical Research Centre. A.P.U. has received funding from the Medical Research Council (MRC) [MR/K501256/1, MR/N013468/1].

## ETHICS STATEMENT

All participants provided written informed consent at the UKBB cohort recruitment. This study received ethical approval from UKBB Ethics Advisory Committee (EAC).

## TRANSPARENCY DECLARATION

The lead author affirms that this manuscript is an honest, accurate, and transparent account of the study being reported; that no important aspects of the study have been omitted. This study was performed under the application of 65 397.

## DISCLAIMER

The views expressed in this article are the personal views of the author(s) and may not be understood or quoted as being made on behalf of or reflecting the position of the regulatory agency/agencies or organizations with which the author(s) is/are employed/affiliated.

## Supporting information


Appendix S1

## Data Availability

Bona fide researchers can apply to use the UK Biobank dataset by registering and applying at http://ukbiobank.ac.uk/register‐apply/. Any additional summary data generated and/or analyzed during the current study are available from the corresponding author on reasonable request.
