# Supplementary material for: Genetic risk and incident venous thromboembolism in middle‐aged and older adults following COVID‐19 vaccination
Source: J Thromb Haemost. 2022 Oct 5;20(12):2887–95. doi: 10.1111/jth.15879 (PMC9538420; doi:10.1111/jth.15879)
Supplement: Supplementary file 1 — Appendix S1 [file JTH-20-2887-s001.docx]

**Supplementary materials:**

**sFigure 1: Distribution of polygenic risk score for venous thromboembolism**

| **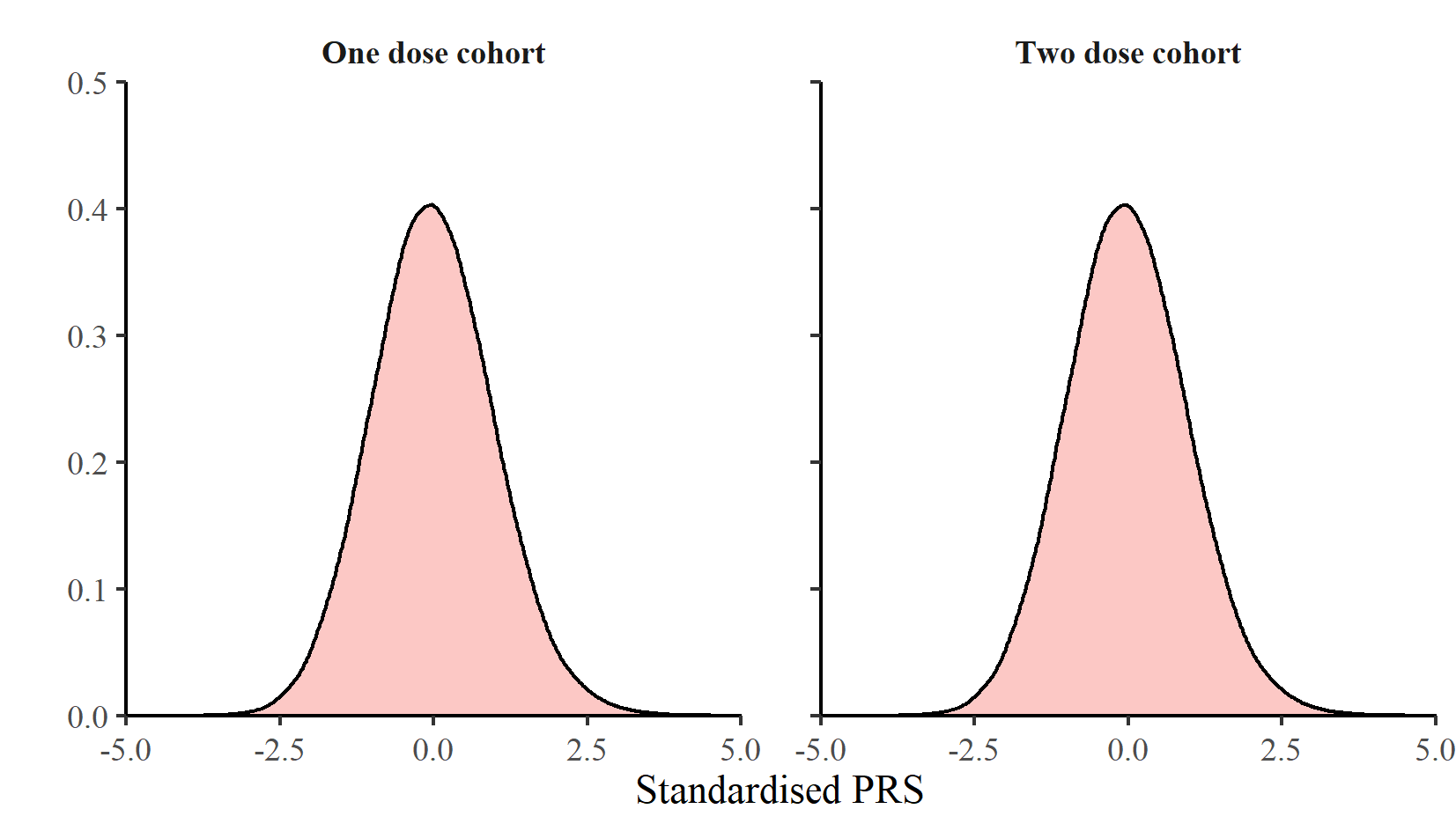** |
| --- |

**sFigure 2:** **The relationship between the PRS value and the risk (hazard ratio) of VTE.**

| 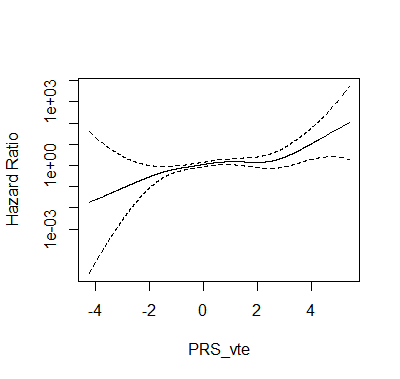  28 days after one dose | 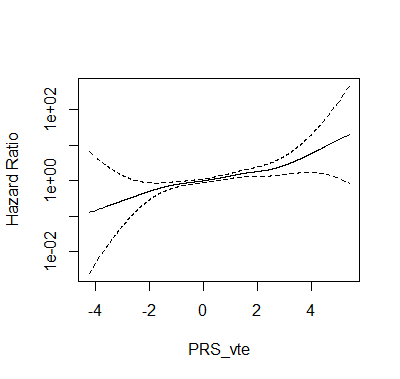  90 days after one dose |
| --- | --- |
| 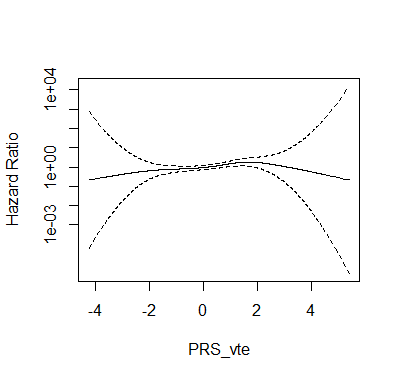  28 days after two doses | 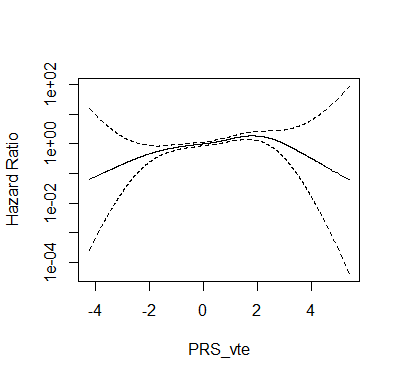  90 days after two doses |

**sTable 1: Summary statistics for candidate SNPs used for constructing the main polygenic risk score (Main PRS_VTE).**

| **Chr:Pos (GRCh37)** | **Rsid** | **Effect Allele** | **Beta** |
| --- | --- | --- | --- |
| 1:11910677 | rs632793 | G | 0.0466 |
| 1:150388318 | rs698915 | A | 0.0561 |
| 1:166533517 | rs764024 | T | 0.0502 |
| 1:168406710 | rs111414961 | A | 0.1183 |
| 1:168500034 | rs72703796 | G | 0.2014 |
| 1:168572720 | rs72705895 | T | 0.1313 |
| 1:168577239 | rs12567872 | T | 0.0893 |
| 1:168619548 | rs1933116 | T | 0.1724 |
| 1:168673803 | rs611769 | C | 0.0562 |
| 1:168714137 | rs10918970 | T | 0.0572 |
| 1:168729611 | rs12117978 | A | 0.2166 |
| 1:168812398 | rs34258243 | G | 0.1015 |
| 1:168889373 | rs78516619 | G | 0.6841 |
| 1:168909952 | rs7526462 | T | 0.0714 |
| 1:168937072 | rs113123846 | G | 0.4417 |
| 1:168960286 | rs1322487 | A | 0.046 |
| 1:168969254 | rs35990973 | A | 0.1057 |
| 1:169012124 | rs1320969 | G | 0.0478 |
| 1:169014610 | rs116140155 | A | 0.2498 |
| 1:169031755 | rs113079063 | T | 0.2356 |
| 1:169038856 | rs12747018 | T | 0.0717 |
| 1:169064630 | rs1200118 | G | 0.1111 |
| 1:169070213 | rs1892091 | C | 0.0526 |
| 1:169079419 | rs1358714 | A | 0.0444 |
| 1:169086013 | rs2143289 | T | 0.2335 |
| 1:169107377 | rs7520186 | T | 0.0873 |
| 1:169276816 | rs10732287 | T | 0.0916 |
| 1:169316610 | rs10158131 | G | 0.0673 |
| 1:169318242 | rs72702145 | A | 0.1342 |
| 1:169463296 | rs147474835 | G | 0.12 |
| 1:169463519 | rs4656683 | T | 0.1781 |
| 1:169486141 | rs966751 | G | 0.1142 |
| 1:169511555 | rs6032 | T | 0.1351 |
| 1:169593113 | rs3917862 | G | 0.2198 |
| 1:169620673 | rs12075684 | G | 0.0534 |
| 1:169659128 | rs185120584 | T | 0.5696 |
| 1:170051841 | rs115476742 | C | 0.0857 |
| 1:170080014 | rs12128208 | C | 0.1373 |
| 1:170174298 | rs12144655 | A | 0.0893 |
| 1:181031783 | rs3936939 | A | 0.0458 |
| 1:201882277 | rs2644120 | C | 0.058 |
| 1:207282149 | rs2842700 | A | 0.1138 |
| 1:207285043 | rs577695638 | C | 0.1708 |
| 1:230417394 | rs3088075 | T | 0.0798 |
| 1:248028780 | rs79322592 | A | 0.0517 |
| 1:248039451 | rs3811444 | C | 0.0513 |
| 1:9341786 | rs677665 | T | 0.0521 |
| 2:127934102 | rs10198483 | A | 0.0559 |
| 2:127979335 | rs111736896 | A | 0.0717 |
| 2:128133076 | rs35293119 | G | 0.0442 |
| 2:128175875 | rs1799809 | G | 0.072 |
| 2:128187428 | rs67495946 | C | 0.0631 |
| 2:128398892 | rs67076363 | A | 0.0733 |
| 2:161845453 | rs75145714 | C | 0.1264 |
| 2:178238316 | rs4893934 | G | 0.0449 |
| 2:198894550 | rs535527575 | G | 0.1319 |
| 2:198946551 | rs6434955 | G | 0.0587 |
| 2:68530180 | rs13013670 | T | 0.0485 |
| 2:68619981 | rs1867312 | C | 0.0585 |
| 3:126238371 | rs11721316 | A | 0.061 |
| 3:126276710 | rs12636448 | C | 0.0456 |
| 3:150872174 | rs75347181 | A | 0.1323 |
| 3:150880908 | rs3116549 | C | 0.0522 |
| 3:185234111 | rs115110838 | T | 0.0649 |
| 3:194787481 | rs116764841 | T | 0.0661 |
| 3:36140951 | rs113114306 | T | 0.1047 |
| 3:39240559 | rs7622284 | T | 0.0741 |
| 3:6084766 | rs11712865 | G | 0.0799 |
| 3:77048575 | rs6799348 | G | 0.1488 |
| 3:88354842 | rs1841009 | T | 0.0567 |
| 3:89250573 | rs7374904 | A | 0.0751 |
| 3:89679389 | rs141912156 | T | 0.1656 |
| 3:90390344 | rs9858006 | A | 0.0465 |
| 3:93518545 | rs139156297 | T | 0.1588 |
| 3:93580976 | rs9290378 | T | 0.0509 |
| 3:94209055 | rs9290227 | T | 0.0849 |
| 4:155251071 | rs116333064 | A | 0.1157 |
| 4:155424231 | rs4323084 | T | 0.1012 |
| 4:155432944 | rs4547780 | G | 0.064 |
| 4:155450158 | rs13435192 | T | 0.1124 |
| 4:155474683 | rs115999709 | G | 0.1456 |
| 4:155494926 | rs79726896 | G | 0.1598 |
| 4:155513276 | rs2070008 | T | 0.0975 |
| 4:155515486 | rs1984906 | G | 0.0671 |
| 4:155538470 | rs13130318 | G | 0.1984 |
| 4:155542926 | rs59234924 | G | 0.1077 |
| 4:155544958 | rs72681247 | T | 0.2163 |
| 4:155553541 | rs79201245 | C | 0.1462 |
| 4:187114479 | rs10866290 | C | 0.0464 |
| 4:187130620 | rs72646294 | A | 0.1947 |
| 4:187136519 | rs113435394 | C | 0.2091 |
| 4:187161283 | rs551251712 | C | 0.2385 |
| 4:187169469 | rs2203111 | G | 0.0901 |
| 4:187178014 | rs3775303 | T | 0.1486 |
| 4:187180115 | rs4253333 | G | 0.0494 |
| 4:187190285 | rs925452 | A | 0.2681 |
| 4:187196853 | rs4253414 | C | 0.1544 |
| 4:187200550 | rs56810541 | T | 0.1975 |
| 4:187205929 | rs4253425 | C | 0.2399 |
| 4:187213360 | rs72712610 | A | 0.1509 |
| 4:187219599 | rs56379917 | A | 0.1638 |
| 4:187220894 | rs13126546 | T | 0.1142 |
| 4:187234735 | rs72712626 | G | 0.1365 |
| 4:187240280 | rs13137269 | T | 0.0617 |
| 4:187267791 | rs75440104 | C | 0.1177 |
| 4:187347217 | rs7685922 | C | 0.054 |
| 4:79917638 | rs140303646 | T | 0.218 |
| 5:143119511 | rs325247 | C | 0.0439 |
| 5:172785895 | rs12234072 | G | 0.0661 |
| 5:32831939 | rs12656497 | T | 0.0438 |
| 5:38708554 | rs16867574 | C | 0.059 |
| 5:63916834 | rs4700642 | A | 0.0491 |
| 5:75992254 | rs56347914 | C | 0.0843 |
| 6:147701133 | rs9373523 | G | 0.06 |
| 6:169633335 | rs11759438 | C | 0.0436 |
| 6:25531133 | rs214057 | C | 0.0446 |
| 6:28436060 | rs2531815 | T | 0.047 |
| 6:29894392 | rs1627764 | G | 0.0525 |
| 6:30639412 | rs3094094 | A | 0.0793 |
| 6:31092767 | rs3095304 | T | 0.0634 |
| 6:31239869 | rs2074492 | T | 0.051 |
| 7:151028181 | rs11981586 | C | 0.0657 |
| 7:157763424 | rs1347390 | G | 0.0515 |
| 8:102881195 | rs118105926 | A | 0.1379 |
| 8:106573309 | rs7341574 | T | 0.0452 |
| 8:106590705 | rs4541868 | C | 0.0876 |
| 8:108291878 | rs4236786 | C | 0.0512 |
| 8:108340982 | rs7004172 | G | 0.051 |
| 8:108347806 | rs6991048 | T | 0.0904 |
| 8:27810577 | rs2685413 | G | 0.0608 |
| 8:27820792 | rs10087301 | A | 0.0632 |
| 8:30265541 | rs117564659 | G | 0.1314 |
| 8:53204323 | rs138757339 | C | 0.2102 |
| 8:78572803 | rs17383689 | G | 0.0833 |
| 8:87143573 | rs7812868 | C | 0.0621 |
| 9:116253293 | rs189064188 | T | 0.0804 |
| 9:124362398 | rs146383320 | T | 0.1278 |
| 9:135985796 | rs3761824 | C | 0.0538 |
| 9:136025460 | rs3888561 | C | 0.0559 |
| 9:136031918 | rs7027827 | A | 0.0729 |
| 9:136056956 | rs10793953 | G | 0.0496 |
| 9:136062437 | rs10441806 | C | 0.105 |
| 9:136069931 | rs7039497 | G | 0.1081 |
| 9:136077004 | rs11244032 | C | 0.0837 |
| 9:136080512 | rs149189328 | C | 0.2669 |
| 9:136081319 | rs11244035 | T | 0.2223 |
| 9:136098498 | rs28470788 | T | 0.0723 |
| 9:136121303 | rs7855466 | T | 0.1054 |
| 9:136124590 | rs78755596 | A | 0.2855 |
| 9:136128731 | rs11244051 | A | 0.384 |
| 9:136144593 | rs66697526 | G | 0.1399 |
| 9:136145404 | rs9411377 | A | 0.308 |
| 9:136152070 | rs8176634 | G | 0.1117 |
| 9:136152722 | rs8176630 | C | 0.1382 |
| 9:136156064 | rs55988407 | G | 0.1664 |
| 9:136156230 | rs78590974 | T | 0.2221 |
| 9:136157037 | rs557317 | A | 0.1499 |
| 9:136177993 | rs4962043 | G | 0.0607 |
| 9:136184782 | rs9411395 | G | 0.1408 |
| 9:136184985 | rs11791119 | T | 0.1052 |
| 9:136185324 | rs11789139 | G | 0.1669 |
| 9:136193356 | rs76771223 | G | 0.1431 |
| 9:136212168 | rs117119759 | A | 0.2099 |
| 9:136226421 | rs141397052 | G | 0.2259 |
| 9:136240304 | rs3124755 | C | 0.1278 |
| 9:136255149 | rs62575992 | C | 0.0967 |
| 9:136268084 | rs3124747 | A | 0.0757 |
| 9:136270538 | rs41302673 | G | 0.242 |
| 9:136277854 | rs2285488 | G | 0.1007 |
| 9:136296530 | rs149181677 | T | 0.2461 |
| 9:136311017 | rs652600 | A | 0.0992 |
| 9:136323826 | rs3094373 | A | 0.2432 |
| 9:136343647 | rs3094326 | G | 0.1027 |
| 9:136359182 | rs736418 | G | 0.0556 |
| 9:136365146 | rs28615587 | T | 0.078 |
| 9:136382716 | rs9802874 | A | 0.0636 |
| 9:136390015 | rs13300181 | A | 0.0616 |
| 9:136397195 | rs11507716 | T | 0.0802 |
| 9:136509514 | rs1611128 | G | 0.0495 |
| 10:121010256 | rs10886430 | G | 0.1289 |
| 10:32397591 | rs211416 | T | 0.0684 |
| 10:45632668 | rs2211163 | A | 0.0724 |
| 10:71144324 | rs2305196 | A | 0.0555 |
| 10:71148728 | rs12416320 | G | 0.1553 |
| 10:71153882 | rs3793846 | T | 0.0572 |
| 10:71181371 | rs36054387 | C | 0.0729 |
| 10:71215107 | rs137936874 | G | 0.2231 |
| 10:71218059 | rs10998791 | A | 0.0677 |
| 10:71245276 | rs78707713 | T | 0.2414 |
| 10:71262048 | rs1665581 | G | 0.0771 |
| 10:71333897 | rs12785008 | C | 0.0691 |
| 10:71346272 | rs12783163 | A | 0.0596 |
| 10:76189250 | rs140438685 | A | 0.2024 |
| 10:80898969 | rs1769758 | G | 0.0436 |
| 10:96011865 | rs1547643 | G | 0.0476 |
| 11:126300537 | rs11600151 | T | 0.0793 |
| 11:32967270 | rs563259534 | T | 0.1048 |
| 11:33247621 | rs2061997 | T | 0.049 |
| 11:46559730 | rs11038913 | T | 0.1126 |
| 11:46745003 | rs5896 | T | 0.0668 |
| 11:46893108 | rs2306029 | T | 0.0641 |
| 11:46896126 | rs72897640 | T | 0.0672 |
| 11:47373425 | rs2856656 | C | 0.3976 |
| 11:47794348 | rs34953939 | A | 0.0931 |
| 11:48865680 | rs369876615 | C | 0.1184 |
| 11:50242788 | rs117653193 | T | 0.1049 |
| 11:51282525 | rs11606922 | C | 0.1115 |
| 11:55436134 | rs72910502 | C | 0.1161 |
| 11:56526894 | rs543926510 | C | 0.1339 |
| 11:56735815 | rs75348906 | A | 0.0695 |
| 11:56875074 | rs141798115 | T | 0.2207 |
| 11:61489705 | rs198428 | A | 0.0464 |
| 11:61571348 | rs174548 | C | 0.0842 |
| 11:61621611 | rs73487492 | A | 0.1034 |
| 11:73283937 | rs12274057 | T | 0.0751 |
| 12:104147207 | rs3751198 | G | 0.0491 |
| 12:111932800 | rs7137828 | C | 0.0424 |
| 12:123861452 | rs28413626 | G | 0.0739 |
| 12:32844798 | rs61926202 | A | 0.2109 |
| 12:39156743 | rs137870902 | T | 0.2329 |
| 12:54734289 | rs11170877 | A | 0.0696 |
| 12:6071943 | rs139727584 | C | 0.1076 |
| 12:6150824 | rs183356 | A | 0.1405 |
| 12:6160614 | rs7135039 | T | 0.0781 |
| 12:6170645 | rs78915411 | G | 0.0823 |
| 13:113787459 | rs3211752 | G | 0.06 |
| 13:113808274 | rs12858483 | G | 0.0588 |
| 14:100108918 | rs12886724 | G | 0.0501 |
| 14:26690604 | rs77398404 | T | 0.0794 |
| 14:66082793 | rs2229678 | C | 0.1842 |
| 14:83281618 | rs112089121 | G | 0.0752 |
| 14:92217670 | rs61988257 | A | 0.0603 |
| 14:94838142 | rs112635299 | T | 0.1463 |
| 15:43757184 | rs190543502 | T | 0.1973 |
| 15:43911751 | rs115384559 | T | 0.3853 |
| 15:44880783 | rs148770227 | C | 0.1837 |
| 15:65114833 | rs35204896 | G | 0.0738 |
| 15:66430422 | rs74245462 | T | 0.0503 |
| 15:96125226 | rs17502085 | A | 0.0525 |
| 16:1405044 | rs116468525 | A | 0.2059 |
| 16:81840709 | rs34603417 | A | 0.0456 |
| 16:81844607 | rs9937779 | C | 0.0568 |
| 16:81870969 | rs12445050 | T | 0.1248 |
| 16:81874200 | rs61374069 | A | 0.0524 |
| 16:81896523 | rs55909816 | C | 0.0437 |
| 16:81902990 | rs4889419 | G | 0.0575 |
| 16:81915832 | rs11150422 | G | 0.053 |
| 16:81971403 | rs1071644 | C | 0.0475 |
| 16:81976177 | rs16956040 | C | 0.0613 |
| 16:89265466 | rs12926888 | A | 0.0449 |
| 17:1966457 | rs1048483 | T | 0.0616 |
| 17:2172753 | rs216181 | A | 0.072 |
| 17:43758898 | rs57222984 | G | 0.0522 |
| 17:67081278 | rs77542162 | G | 0.1932 |
| 17:7785590 | rs78209469 | T | 0.0867 |
| 17:8393900 | rs2270744 | C | 0.0447 |
| 18:74465347 | rs62112094 | A | 0.059 |
| 18:75283432 | rs2032276 | G | 0.0435 |
| 18:8800723 | rs631126 | C | 0.0579 |
| 19:10639312 | rs8100818 | T | 0.0483 |
| 19:10659971 | rs11668544 | G | 0.1314 |
| 19:10688153 | rs187758170 | A | 0.1465 |
| 19:10734951 | rs8110479 | C | 0.1131 |
| 19:10741622 | rs12710257 | G | 0.1164 |
| 19:10898413 | rs8107372 | T | 0.0515 |
| 19:17004049 | rs1054533 | T | 0.0451 |
| 19:33896432 | rs4805881 | C | 0.0544 |
| 19:3464793 | rs142170418 | C | 0.1574 |
| 19:45426792 | rs141622900 | A | 0.1007 |
| 19:46268902 | rs2341097 | T | 0.0502 |
| 19:49241006 | rs12981072 | C | 0.0469 |
| 19:55536595 | rs1613662 | A | 0.0818 |
| 19:7832001 | rs874492 | A | 0.0481 |
| 20:22672552 | rs6137727 | A | 0.0534 |
| 20:22938940 | rs62204096 | A | 0.1565 |
| 20:23000653 | rs6076004 | T | 0.0472 |
| 20:23077117 | rs149439892 | A | 0.1527 |
| 20:23170450 | rs34397775 | A | 0.084 |
| 20:23182559 | rs6083037 | A | 0.0798 |
| 20:32485961 | rs6087538 | C | 0.0585 |
| 20:32523172 | rs6059574 | G | 0.0479 |
| 20:33434252 | rs56244533 | A | 0.0567 |
| 20:33435161 | rs17092148 | G | 0.0612 |
| 20:33451060 | rs75627267 | T | 0.1209 |
| 20:33587569 | rs551986443 | C | 0.1488 |
| 20:33612647 | rs564783003 | C | 0.1488 |
| 20:33733180 | rs2050652 | G | 0.0564 |
| 20:33762035 | rs2069946 | C | 0.1255 |
| 20:33772243 | rs6060288 | A | 0.1178 |
| 20:33858772 | rs6058218 | G | 0.0552 |
| 20:33895947 | rs6058227 | T | 0.0813 |
| 20:34025983 | rs143383 | G | 0.0482 |
| 20:34562935 | rs185663249 | A | 0.1487 |
| 20:34712310 | rs6141600 | C | 0.0482 |
| 22:42461918 | rs2854827 | G | 0.0589 |
| 22:43107837 | rs9620086 | G | 0.0609 |
| 22:44324730 | rs738408 | C | 0.0566 |
| 1:169519049 | rs6025 | T | 0.4031 |
| 11:46761055 | rs1799963 | A | 0.2741 |

**sTable 2: Summary statistics for candidate SNPs used for constructing sensitive polygenic risk score (Secondary PRS_VTE).**

| **SNPs (GRCh37)** | **Risk Allele** | **Allelic OR** |
| --- | --- | --- |
| rs6025 | T | 3.25 |
| rs4524 | T | 1.20 |
| rs2066865 | A | 1.24 |
| rs4253417 | C | 1.27 |
| rs529565 | C | 1.55 |
| rs1799963 | A | 2.29 |
| rs6087685 | C | 1.15 |
| rs4602861 | A | 1.20 |
| rs78707713 | T | 1.28 |
| rs2288904 | G | 1.19 |

**sTable 3: ICD-10 codes for the identification of venous thromboembolism.**

|  |  |
| --- | --- |
| "I26" | Pulmonary embolism |
| "I260" | Pulmonary embolism with mention of acute cor pulmonale |
| "I269" | Pulmonary embolism without mention of acute cor pulmonale |
| "I801" | Phlebitis and thrombophlebitis of femoral vein |
| "I802" | Phlebitis and thrombophlebitis of other deep vessels of lower extremities |
| "I803" | Phlebitis and thrombophlebitis of lower extremities, unspecified |
| "I81" | Portal vein thrombosis |
| "I82" | Other venous embolism and thrombosis |
| "I820" | Budd-Chiari syndrome |
| "I822" | Embolism and thrombosis of vena cava |
| "I823" | Embolism and thrombosis of renal vein |
| "I828" | Embolism and thrombosis of other specified veins |
| "I829" | Embolism and thrombosis of unspecified vein |

**sTable 4: Association between the polygenic risk score and the negative control outcome.**

|  | **Number of people** | **Number of cases** | **Incidence rate (95% CI)*** | **Adjusted hazard ratio (95% CI) ^$^** |
| --- | --- | --- | --- | --- |
| **Diabetes** |  |  |  |  |
| Whole UKBB (Pre-pandemic) | 373,207 | 2522 | 1.85 (1.78 to 1.93) | 1.02 (0.98 to 1.06) |
| Whole UKBB (Early-pandemic) | 368,112 | 1278 | 1.26 (1.20 to 1.34) | 0.98 (0.93 to 1.04) |
